# Power Calculation for Noninferiority Stepped-Wedge Cluster Randomized Trials

**DOI:** 10.64898/2026.10.04.26364705

**Published:** 2026-10-06

**Authors:** Justin Guo, Chih-Yuan Hsu, Yu Shyr

## Abstract

**Background:**

Stepped-wedge cluster randomized trials (SW-CRTs) are increasingly used when interventions need to be implemented sequentially across clusters. Despite extensive developments in the design and analysis of superiority SW-CRTs, methods for planning noninferiority SW-CRTs remain limited.

**Methods:**

We propose swNIsim, a simulation-based framework for power calculation in noninferiority SW-CRTs using generalized linear mixed-effects models (GLMMs). The proposed approach accommodates both continuous and binary outcomes and supports practical design features, including unequal numbers of clusters across sequences, varying cluster-period sizes, delayed intervention effects, and analyses with or without adjustment for secular time effects. Noninferiority margins can be specified using several commonly applied strategies.

**Results:**

Through extensive simulation studies, we examined the impact of key design parameters on statistical power, including the number of periods, the number of clusters per sequence, the number of individuals per cluster-period, and the degree of between-cluster variability. For a fixed total sample size, designs with more time periods consistently achieved higher power. Increasing the number of clusters per sequence produced modest gains in power, whereas greater between-cluster variability reduced power. Type I error rates were well controlled across the scenarios examined. Balanced allocation of clusters across sequences was more efficient than unbalanced allocation schemes. Two real-world SW-CRT examples were used to illustrate application of the method.

**Conclusions:**

The proposed swNIsim provides a flexible and practical approach for power calculation in noninferiority SW-CRTs. By accommodating a wide range of design configurations and outcome types, and through implementation as a freely available R package, swNIsim may facilitate the planning and design of noninferiority stepped-wedge trials.

## Full Text Availability

The license terms selected by the author(s) for this preprint version do not permit archiving in PMC. The full text is available from the preprint server.

